# 414. Observed Time Burden with Nursing Practices in an Emergency Room COVID-19 Isolation Zone at a University Affiliated Hospital in Korea

**DOI:** 10.1093/ofid/ofab466.614

**Published:** 2021-12-04

**Authors:** Imyoung Choi, JaHyun Kang

**Affiliations:** 1 Seoul National University Hospital, Seodaemungu, Seoul-t’ukpyolsi, Republic of Korea; 2 College of Nursing and Research Institute of Nursing Science, Seoul National University, Seoul, Seoul-t’ukpyolsi, Republic of Korea

## Abstract

**Background:**

The coronavirus disease 2019 (COVID-19) has caused great burdens on emergency room (ER) and front-line ER healthcare personnel faced with great challenges, including threats to their safety. This study aimed to provide a basis for additional workload of ER nurses who are charged with providing care for COVID-19 confirmed or suspicious cases.

Table 1. Summary of Frequency and Time Burden with Nursing Practices in an Emergency Room COVID-19 Isolation Zone. Note. IV, intravenous; IM, intramuscular; ID, intradermal; SC, subcutaneous; PPE, personal protective equipment; CPR, cardiopulmonary resuscitation

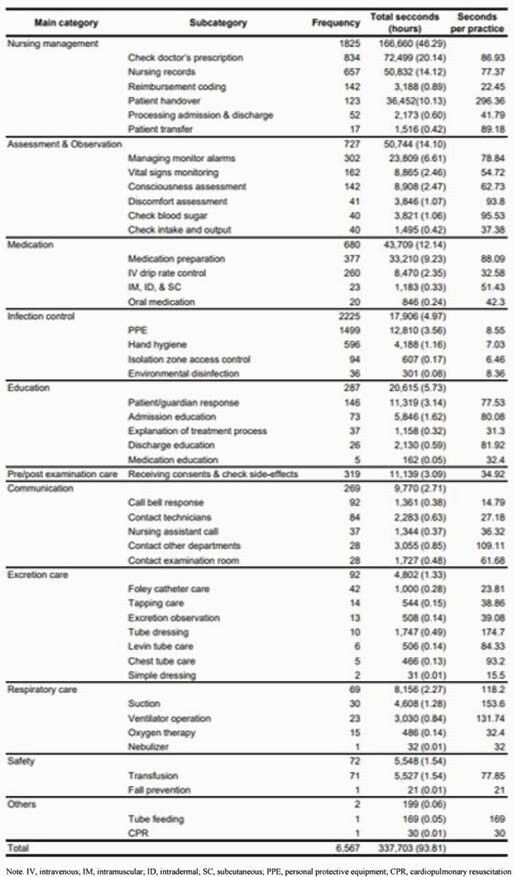

**Methods:**

With institutional review board approval, we recruited ER nurses who were assigned to COVID-19 isolation zone with more than 6 months’ ER work experience. After their demographic information were collected through a questionnaire, their nursing practices and practice time during their 1 shift (day or evening) were recorded by one researcher using a stopwatch and an observation form. For each observation shift, unit-related information was collected, including the numbers of hospitalized patients, admission, discharge, and transfer of patients. For each nursing practice, frequency and total time spent were analyzed using descriptive statistics with SPSS 26.0 program.

**Results:**

From January 4 to February 22, 2021, a total 18 nurses (27.4 years old on average with 25.2 months of ER experience) were observed from 20 different shifts. During the observation period, the average number of nurses’ working hours was 8.27 ± 0.39 hours. A total of 6,567 tasks were monitored with 337,703 seconds (93.81 hours) of the total time spent. Infection control practices were most frequent (33.88%) followed by nursing management (27.80%), assessment and observation (11.07%), medication (10.35%), pre and post examination care(4.86%), education (4.37%), communication (4.10%), safety care (1.10%), and others (0.03; Table 1). Nursing management (e.g., nursing recording) was most time-consuming (49.29%) followed by assessment and observation (15.03%), medication (12.94%), patient education (6.10%), infection control (5.30%), and safety care (1.64%).

**Conclusion:**

This study showed that infection control practices were most frequent while time spent was relatively insignificant among ER nurses in charge of COVID-19 isolation zones. Further studies for more observations or with different study designs at other ER settings are necessary to understand nurse’s burdens with COVID-19 emergency care.

**Disclosures:**

**All Authors**: No reported disclosures

